# FAP+ activated fibroblasts are detectable in the microenvironment of endometriosis and correlate with stroma composition and infiltrating CD8+ and CD68+ cells

**DOI:** 10.1093/hropen/hoaf003

**Published:** 2025-01-24

**Authors:** Franziska Kellers, Ulf Lützen, Frederik Verburg, Annett Lebenatus, Karolin Tesch, Fatih Yalcin, Moritz Jesinghaus, Valentina Stoll, Hanna Grebe, Christoph Röcken, Dirk Bauerschlag, Björn Konukiewitz

**Affiliations:** Department of Pathology, University Hospital Schleswig-Holstein, Kiel, Germany; Department of Nuclear Medicine, Molecular Diagnostic Imaging and Therapy, University Hospital Schleswig-Holstein, Kiel, Germany; Department of Radiology and Nuclear Medicine, Erasmus MC, Rotterdam, The Netherlands; Department of Radiology and Neuroradiology, University Hospital Schleswig-Holstein, Kiel, Germany; Department of Radiology and Neuroradiology, University Hospital Schleswig-Holstein, Kiel, Germany; Department of Pathology, University Hospital Schleswig-Holstein, Kiel, Germany; Institute of Pathology, Phillips University Marburg and University Hospital Marburg, Marburg, Germany; Department of Pathology, University Hospital Schleswig-Holstein, Kiel, Germany; Department of Pathology, University Hospital Schleswig-Holstein, Kiel, Germany; Department of Pathology, University Hospital Schleswig-Holstein, Kiel, Germany; Department of Obstetrics and Gynaecology, Jena University Hospital, Jena, Germany; Department of Pathology, University Hospital Schleswig-Holstein, Kiel, Germany

**Keywords:** immune evasion, fibrosis, PET-CT, stroma, immunosurveillance, endometrioma, fibroblast activation, FAPalpha, macrophages, cytotoxic T-lymphocyte

## Abstract

**STUDY QUESTION:**

Do activated fibroblasts expressing fibroblast activation protein-α (FAP) – which is traceable in positron emission topography/computed topography (PET/CT) – play a role in the microenvironment of endometriosis?

**SUMMARY ANSWER:**

Activated fibroblasts expressing FAP are detectable in endometriotic lesions and correlate with iron and collagen content and infiltrating CD8-positive cytotoxic T cells and CD68-positive macrophages in the microenvironment endometriotic lesions.

**WHAT IS KNOWN ALREADY:**

FAP-positive activated fibroblasts are found in various fibrosis-related pathologies and in the desmoplastic stroma of solid tumours; they can be traced in PET/CT but have not been investigated in the context of endometriosis, a chronic disease involving hormone-mediated repetitive tissue remodelling and fibrosis.

**STUDY DESIGN, SIZE, DURATION:**

We analysed a cohort of endometriosis patients (n = 159) who had undergone surgery with removal of endometriotic foci at our University Hospital (tertiary care centre) between 2018 and 2024. All patients provided written informed consent. The median age of the patients was 34 years. In total, 245 samples from different locations were analysed retrospectively.

**PARTICIPANTS/MATERIALS, SETTING, METHODS:**

We investigated the expression of FAP and its relation to stroma composition and the immune microenvironment of endometriosis in 245 specimens from peritoneal lesions, ovarian endometriomas, deep infiltrating endometriosis, and extra-abdominal lesions using conventional histology and immunohistochemistry followed by digital image analysis. Tissue within a radius of 500 µm of ectopic endometrium-like epithelium was analysed. To measure FAP expression in the perilesional stroma, a histoscore (H-score) was calculated. Masson trichrome staining was used to determine collagen content. Prussian blue staining for iron was used for age-dating of lesions. The abundance of CD68-positive macrophages and CD8-positive cytotoxic T cells within the microenvironment of ectopic endometriotic glands was analysed. Extra-lesional tissue served as controls.

**MAIN RESULTS AND THE ROLE OF CHANCE:**

Distinct FAP expression (H-score >10) was observed in 84% of endometriotic lesions and in only 4% of extra-lesional controls. FAP expression was significantly higher in endometriotic lesions (mean H-score 61.8) than in extra-lesional tissue (mean H-score 3.8, *P* < 0.0001). There was a significant (*P* < 0.05) association with collagen content when comparing samples with low (H-score <100) and high (H-score ≥100) FAP expression, and a significant difference in FAP expression correlating with the tissue iron content when comparing strong staining intensity and negative samples (*P* < 0.0005) or samples with weak staining intensity (*P* < 0.005). Moreover, the abundance of CD8-positive and CD68-positive cells was significantly higher (*P* < 0.0001) in samples with high FAP expression (H-score ≥100).

**LARGE SCALE DATA:**

N/A.

**LIMITATIONS, REASONS FOR CAUTION:**

This study proves the presence of FAP-positive fibroblasts in endometriosis by immunohistological methods. However, to translate targeting FAP into endometriosis diagnostics, these results have to be compared to imaging data and FAP inhibitor (FAPi) PET/CT has to be validated in a structured way on a large patient cohort. Moreover, we show that FAP expression is intertwined with the immune cell infiltrate in the microenvironment of endometriosis. To explore and understand mechanisms contributing to chronic inflammation, immune evasion, and fibrosis, more studies including more immune cell subtypes and functional experiments are needed.

**WIDER IMPLICATIONS OF THE FINDINGS:**

FAP-positive activated fibroblasts not only impact the immune microenvironment of endometriosis and are linked to increased macrophage and cytotoxic T-cell infiltration, as we showed, but could also provide new options for non-invasive diagnostic methods and an improvement of the diagnostic workup prior to surgery. FAPi PET/CT should be considered when exploring new diagnostic options in endometriosis.

**STUDY FUNDING/COMPETING INTEREST(S):**

This work was funded by the Deutsche Forschungsgemeinschaft (DFG, German Research Foundation) ‘Clinician Scientist Program in Evolutionary Medicine’ (project number 413490537 to F.K.). We acknowledge financial support by Land Schleswig-Holstein within the funding programme ‘Open Access Publikationsfonds’. The authors declare that they have no conflicts of interest related to this work.

WHAT DOES THIS MEAN FOR PATIENTS? Endometriosis is a chronic disease affecting ∼10% of the menstruating population. It is caused by tissue that resembles the inner lining of the uterus which is misplaced and grows outside of the uterine cavity. Despite being in a different location, this tissue is still subject to hormonal influences and undergoes monthly cycling, including bleeding and tissue remodelling. Consequently, this repetitive tissue injury leads to inflammation, iron deposits, and scarring of these lesions. This involves connective tissue cells, so-called ‘fibroblasts’, which grow in and replace the wounded tissue. The inflammation and the scarring lead to a variety of symptoms, such as chronic pain and infertility, which can impose a tremendous impairment of the quality of life of patients with endometriosis. The diagnosis of endometriosis is usually established by combining clinical findings and imaging data and sometimes confirmed by surgical removal of the lesions followed by laboratory tests of the tissue. However, due to often non-specific symptoms, as well as a lack of awareness and a lack of specific diagnostic options, the diagnosis is often delayed.

Searching for a new detection method of endometriosis, we came across a molecule called ‘fibroblast activation protein-alpha’ (FAP). FAP recently received much attention in the scientific community because it can be detected in positron emission topography/computed topography (PET/CT), which is a non-invasive imaging method. FAP is known to occur on activated fibroblasts in many malignant tumours and also non-malignant diseases that involve tissue remodelling and scarring.

To explore the occurrence of FAP in endometriosis, we looked at 245 tissue specimens of different locations from a cohort of 159 endometriosis patients. We used a technique called immunohistochemistry to make activated fibroblasts visible in the tissue using antibodies directly binding to FAP. To compare the results, we digitalized the specimens. We compared FAP abundance in endometriotic lesions of various locations with its expression in tissue distant from the lesions (as the control group). Hereby, we found a detectable amount of FAP in 84% of endometriotic lesions and in only 4% of the distant controls. For each of the locations we studied, we could confirm that FAP expression was significantly higher in endometriotic lesions than in ‘normal’ tissue distant from the lesions. To study the role of activated fibroblasts in endometriosis, we explored the composition of the tissue surrounding the endometriotic lesions. We found a connection of FAP expression with tissue iron content which is an indicator of the age of an endometriotic lesion. Moreover, we found that lesions with higher FAP expression also had a higher collagen content. Since activated fibroblasts are known to interact with the immune system, we explored a connection between FAP-positive activated fibroblasts and the number of infiltrating immune cells in endometriotic lesions. We found higher numbers of both macrophages, that play an important role in tissue remodelling, and CD8-positive T cells, a subpopulation of lymphocytes important for an effective immune response.

Our results prove for the first time that ongoing hormonal cycling which leads to chronic inflammation and fibrosis in endometriosis involves fibroblast activation. This does not only impact the microenvironment surrounding endometriotic lesions, as we showed, but may also provide new options for endometriosis diagnostics in the future.

## Introduction

Endometriosis is a chronic gynaecological disease affecting an estimated 10% of the menstruating population and up to 50% of infertile women (Zondervan *et al.*, 2020). Endometriosis is defined by ectopic endometrial tissue often associated with an inflammatory process (International Working Group of AAGL, ESGE, ESHRE and WES *et al.*, 2021), which undergoes cyclic hormone-mediated changes and tissue remodelling leading to a vast variety of clinical presentations and symptoms, including chronic pelvic pain, dyspareunia, and infertility. Additionally, endometriosis is associated with an increased risk for certain types of cancer including endometriosis-associated ovarian cancer (Buis *et al.*, 2013; Al-Badawi *et al.*, 2024). Diagnosis is based on the clinical presentation and physical examination in combination with imaging such as ultrasound and magnetic resonance imaging if deep infiltrating endometriosis is suspected. While the ESHRE 2022 guideline (Becker *et al.*, 2022) recommends a diagnosis of endometriosis based on clinical examination in combination with imaging results (followed by empiric hormonal therapy in symptomatic patients), the Sk2-guideline of the German, Austrian and Swiss Society of Gynaecology and Obstetrics (DGGG, OEGGG and SGGG), which is valid until 08/2025 (Burghaus *et al.*, 2021), recommends diagnostic laparoscopy and excision of lesions followed by histological analyses of the obtained tissue samples as the gold standard in diagnostics of endometriosis. Due to non-specific symptoms and varying clinical presentation, as well as a lack of biomarkers, awareness, and specific non-invasive diagnostics, the diagnosis is often delayed for years.

In the environment of endometriotic lesions, fibrosis plays a key role and is highly related to the endometriosis-associated morbidity and disease presentation (Vigano *et al.*, 2018). Often, endometriotic lesions consist largely of fibrotic tissue, with ectopic endometrioid glands and endometrioid stroma often constituting only a minor component of the lesions (Donnez *et al.*, 1996; Muzii *et al.*, 2007; Somigliana *et al.*, 2012). A cell type relevant to fibrotic conditions that has recently gained more attention are activated fibroblasts. Activated fibroblasts occur rarely in physiological settings but frequently in disease contexts involving tissue remodelling and fibrosis (Hamson *et al.*, 2014; Fitzgerald and Weiner, 2020). Moreover, activated fibroblasts are part of the microenvironment of a multitude of solid tumours (Garin-Chesa *et al.*, 1990), where cancer-associated fibroblasts (CAFs) are associated with decreased tumour immunosurveillance (Joyce and Fearon, 2015; Lakins *et al.*, 2018) and a negative prognosis (MacNeil *et al.*, 2021). CAFs influence the extracellular matrix structure by paracrine signalling (Lee *et al.*, 2011; Qi *et al.*, 2022). Activated fibroblasts can be identified by their expression of FAP, a type II transmembrane serine protease. Interestingly, FAP can be traced by an FAP inhibitor (FAPi) and visualized in positron emission topography/computed topography (PET/CT) (Kratochwil *et al.*, 2019) and therefore provides a diagnostic and potentially therapeutic target. FAPi PET/CT has been explored in gynaecological malignancies (Dendl *et al.*, 2021, 2023) but has not yet been evaluated for diagnostics of endometriosis; in contrast, fibrotic diseases such as endometriosis have merely been noted for creating artefacts in malignancy diagnostics (Lakhani *et al.*, 2015).

Therefore, the aim of this study was to explore the presence and role of activated fibroblasts in the microenvironment of endometriosis with regard to FAP expression, stroma composition, and immune cell infiltrate in order to broaden the understanding of the disease and to explore further diagnostic options such as FAPi PET/CT targeted at activated fibroblasts in the stroma. This included an assessment of perilesional FAP expression in a large patient cohort on samples of different locations, for which a digital image analysis workflow was established. Moreover, this study covers an analysis of a relation of the abundance of activated fibroblasts with extracellular matrix composition, age-dependent iron deposits, and infiltrating immune cells.

## Materials and methods

### Patient cohort

All patients provided written informed consent. All patients underwent surgery for removal of endometriotic lesions at the University Hospital Schleswig-Holstein (UKSH) Campus Kiel between 2018 and 2024. A total of 245 samples from 159 patients were analysed (Table 1). If more than one sample per patient was used, the samples were retrieved from different locations. The median patient age was 34 years (mean 34.97 years, range 18–69 years). Besides establishing a diagnosis of endometriosis, surgical intervention was indicated in our patient cohort due to the presence of one or more of the following: severe symptoms necessitating radical excision of lesions in 59% of cases; symptomatic or suspect ovarian cysts or abdominal masses in 34% of cases; adhesions or complications of endometriosis in 15% of cases; and/or indications unrelated to endometriosis symptoms, such as visceral surgeries with an incidental discovery of endometriotic lesions in 3% of cases. Of the patients, 34% were on hormonal treatment at the time of surgery. Deep infiltrating endometriosis was present or suspected in 47% of patients. Adenomyosis uteri was present or suspected in 34% of patients. Patients with overlapping pathologies such as associated ovarian carcinoma in the same specimen were excluded from the study.

**Table 1.** Overview of the tissue samples eligible for histological and immunohistochemical analyses.

Analysis	Total	FAP low	FAP high	**FAP<10** *	**FAP≥10** *
**FAP**	245	189	56	39	206
Ovarian endometriosis	38	21	17	8	30
Peritoneal lesions	167	137	30	26	141
Deep infiltrating endometriosis	32	26	6	4	28
Extra-abdominal endometriosis	8	5	3	1	7
Extra-lesional (number of pairs)	234	234	0	224	10
**Collagen**	219	166	53		
**Iron**	243				
Negative	32				
Fe low	68				
Fe high	143				
**CD68**	242	187	55		
**CD8**	243	189	54		

* H-score.

FAP, fibroblast activation protein-α; Fe, iron; H-score, histoscore.

### Ethics

All patients had provided written informed consent. This study was approved by the Committee of Ethics of the Medical Faculty of the Christian Albrechts Universität zu Kiel (Vote No. D 643/23) and is in accordance with the Helsinki Declaration of 1964 and its later amendments.

### Histology and immunohistochemistry

Tissue was fixed with 4% formaldehyde before embedding in paraffin. Tissue sections were cut into 5 µm thin sections using a microtome; the tissue sections were deparaffinized and rehydrated in water. Haematoxylin and eosin (H&E) staining was carried out on a Tissue Tek Prisma^®^ Plus autostainer (Sakura Finetek Germany GmbH, Umkirch, Germany). After review of the H&E-stained slides by board-certified surgical pathologists, further analyses on consecutive slides were conducted.

Prussian blue staining was performed according to Perls’ reaction (Perls, 1867). After deparaffinizing, the tissue sections were stained with 0.08% potassium hexacyanoferrate (7 min, Sigma-Aldrich Chemie GmbH, Taufkirchen, Germany, Catalogue No. 104984). Counterstaining was performed with aluminium-sulphate solution (10 min, Diagonal GmbH & Co. KG, Münster, Germany, Catalogue No. 50103191).

For Masson Goldner trichrome analysis, tissue sections were deparaffinized and stained with haematoxylin solution (5 min, Sigma-Aldrich Chemie GmbH, Catalogue No. 1.05175.0500), Goldner1 (10 min, Dr K. Hollborn & Söhne GmbH & Co. KG, Leipzig, Germany, Catalogue No. G19-1000), Goldner2 (8 min, Dr K. Hollborn & Söhne GmbH & Co. KG, Catalogue No. G20-1000), and Goldner3 (6 min, Dr K. Hollborn & Söhne GmbH & Co. KG, Catalogue No. G21-1000) solution.

For immunohistochemistry, sections were stained with antibodies directed against FAP, CD8, CD68, and CD10 (Supplementary Table S1). Antigen retrieval was achieved with ER2 (EDTA-buffer Bond pH 9.0; 20 min). The immunoreaction was visualized with the Bond™ Polymer Refine Detection Kit (DS 9800; brown labelling; Novocastra; Leica Biosystems Melbourne Pty Ltd, Mount Waverley, Australia) resulting in a brown colour, or with the Bond™ Polymer Refine Detection Kit (DS 9390; red labelling; Novocastra; Leica Biosystems Melbourne Pty Ltd) resulting in a red colour, and counterstained with haematoxylin. IHC was carried out on the autostainer BOND™ RX system (Leica Biosystems Melbourne Pty Ltd, Mount Waverley, Australia). All antibodies used in this study have been validated and controlled for specificity using internal and external positive and negative controls. The stained tissue sections were digitalized using a NanoZoomer S60 Digital slide scanner (Hamamatsu Photonics K.K., Hamamatsu City, Japan) at 40× magnification.

### Digital image analysis

For digital image analysis, open-source software QuPath (Bankhead *et al.*, 2017) version 5.0 was used. Ectopic endometriotic tissue was manually annotated. Annotations within one image were merged and expanded by 500 µm to capture the adjacent, lesion-surrounding stroma. For analysis of CD68, the interior of the annotation (lumen of the endometriotic glands) was included in the analysis. In the expanded area, tissue was detected by pixel classification. To measure FAP expression, cell detection using the StarDist extension (Uwe Schmidt, 2018) was followed by QuPath’s built-in cell intensity classification (Bankhead *et al.*, 2017) using a three-tiered expression cut-off determined by visual inspection. For FAP, a histoscore (H-score) with a range from 1 to 300 was calculated based on the percentage of stained cells and the staining intensity (H-score = 1× % weakly positive cells + 2× % moderately positive cells + 3× % strongly positive cells). CD8- and CD68-positive cells were detected by using QuPath’s built-in cell detection algorithms (Bankhead *et al.*, 2017). For CD8 and CD68, a single threshold was defined and the number of positive cells per mm^2^ was obtained. Extra-lesional tissue was annotated and processed in the same way to serve as controls. For Masson Goldner trichrome analysis, stain separation was conducted on digitalized slides using QuPath colour deconvolution according to Ruifrok and Johnston (2001). Pixel classification was then used on the light green channel to determine the percentage of tissue area above threshold which was determined as collagen. Scripts, classifier settings, and cut offs are available from the corresponding author upon request.

### Statistics

For data analysis and statistics, Microsoft Excel 2019 and GraphPad Prism (version 10.2, GraphPad Software Inc., San Diego, CA, USA) were used. Data of two groups were analysed using Student’s *t*-test. Student’s *t*-tests were performed according to an unpaired, two-tailed normal distribution of values (95% CI, definition of significance: *P* < 0.05). Paired two-tailed Student’s *t*-tests were used to compare different features of the same sample; unpaired Student’s *t*-tests were performed according to a normal distribution of values (95% CI, definition of significance: *P* < 0.05). Nested *t*-test was used to compare nested data. Ordinary one-way ANOVA was used when three or more groups were compared. Unpaired *t*-test was used to study subgroups of the cohort. Pearson’s correlation coefficient was calculated to analyse correlation of marker expression. Fisher’s exact test was used when comparing two categorial factors. Data are shown as mean ± 95% CI.

## Results

### FAP immunohistochemistry

**Figure 1.**
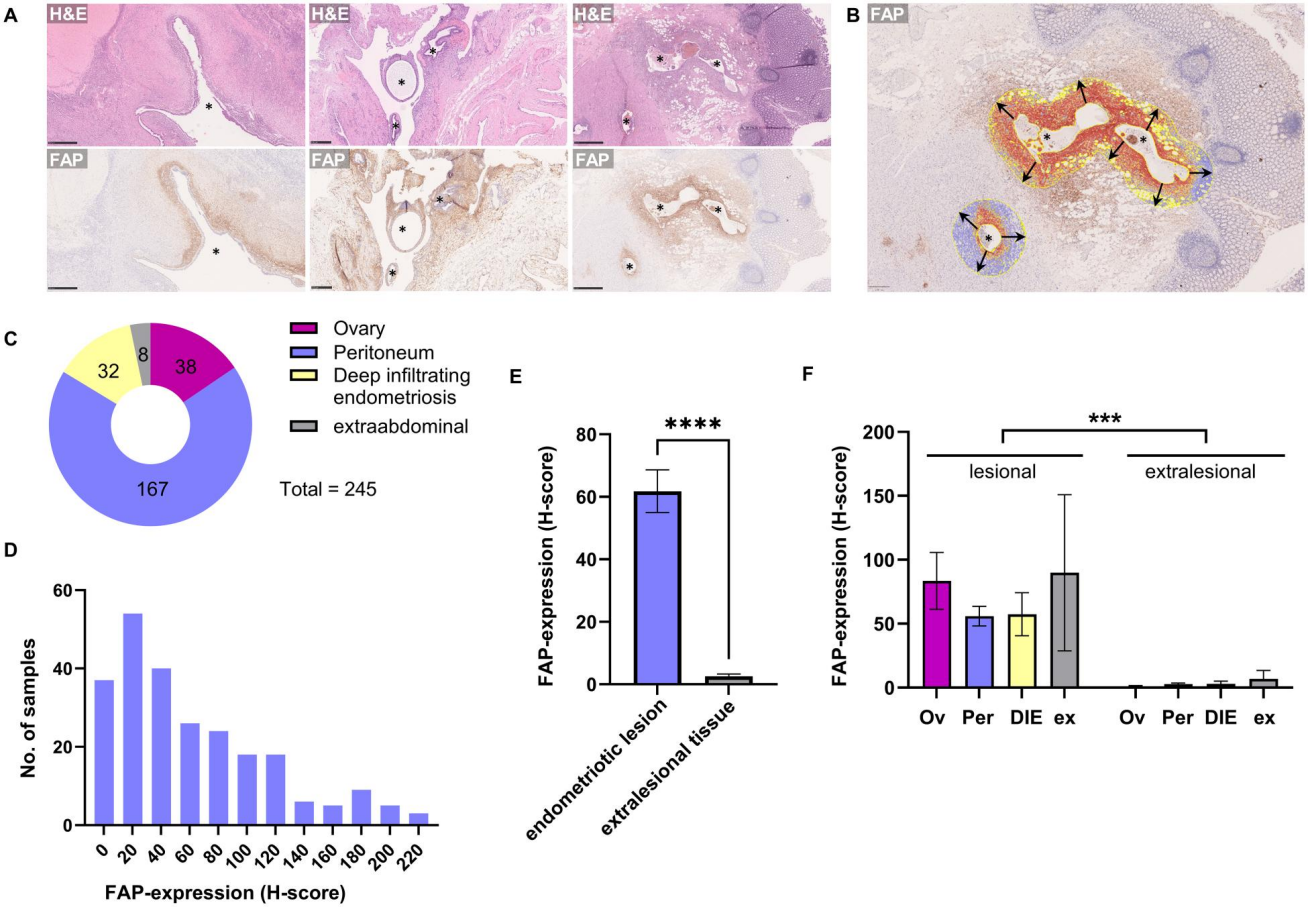
**Analysis of fibroblast activation protein-α (FAP) expression in endometriotic lesions and extralesional tissue using immunohistochemistry and digital image analysis.** (**A**) Haematoxylin and eosin-stained sections (upper panel) and corresponding FAP immunohistochemistry (lower panel) of ovarian endometrioma (left, scale bar 500 µm), peritoneal superficial endometriosis (middle, scale bar 250 µm), and deep infiltrating endometriosis of the rectum (right, scale bar 1000 µm); lesions depicted by asterisks. (**B**) Digital image analysis (QuPath) for assessment of staining intensity and percentage of positive cells in close distance to lesions in deep infiltrating endometriosis of the rectum. Ectopic endometrioid cystic glands (asterisk) are annotated and the annotation is expanded by 500 µm (arrows). In the expanded area, tissue detection (yellow lines) is followed by cell detection and intensity classification. The overlay depicts cell intensity classification: *blue* negative, *yellow* weak positivity, *orange* moderate positivity, *red* strong positivity. Scale bar: 400 µm. (**C**) Localization distribution of analysed samples. (**D**) Histogram of FAP expression (histoscore [H-score]) of the lesional samples. (**E, F**) FAP expression (H-score) of lesional and extralesional control tissue. Ov, ovary; Per, peritoneum; DIE, deep infiltrating endometriosis; ex, extra-abdominal. Data are shown as mean with 95% CI. ***P < 0.0005, ****P < 0.0001, two-tailed paired *t*-test (E) and nested *t*-test (F). Comparisons indicated by horizontal lines.

Due to the fibrotic and chronic inflammatory nature of endometriosis, we hypothesized that activated fibroblasts might play a role in the microenvironment of endometriosis. To explore their role and potential suitability as a target for FAPi PET/CT in future studies, we assessed their abundance immunohistochemically (Fig. 1A). We analysed 245 samples (Table 1) from different locations which were grouped into ovarian endometriomas, superficial peritoneal lesions, deep infiltrating endometriosis, and extra-abdominal specimens (Fig. 1C).

As endometriosis involves tissue of various locations, specimens vary greatly in size and tissue composition which makes objective quantification on larger slides challenging. Thus, we needed an objective approach to quantify FAP expression specifically in close distance to endometriotic lesions on whole slides. We established a digital image analysis workflow using QuPath (Bankhead *et al.*, 2017) to determine the lesion-adjacent perilesional stroma surrounding ectopic endometrioid glands within a distance of 500 µm (Fig. 1B) and calculated a H-score factoring in both the percentage of FAP-positive fibroblasts and the staining intensity. To compare the endometriosis-related stroma with non-pathological tissue from the same site, extralesional areas were annotated and served as a control group.

Elevated FAP expression (H-score ≥10) was detectable in 84% of endometriosis cases in endometriomas of the ovary, superficial peritoneal lesions, deep infiltrating endometriosis, and extra-abdominal lesions (Fig. 1A, C, D, and F) and only 4% of extralesional controls. There was significantly higher (*P* < 0.0001) FAP expression in the lesional tissue (mean H-score 61.8) than in extra-lesional control tissue (mean H-score 3.8, Fig. 1E). A nested analysis of the location subgroups revealed that the higher expression in endometriotic lesions remained significant when comparing each of the examined locations separately (*P* < 0.0005, Fig. 1F). There was no significant association of FAP expression with hormonal treatment at the time of surgery (*P* > 0.05).

### Stroma composition

**Figure 2.**
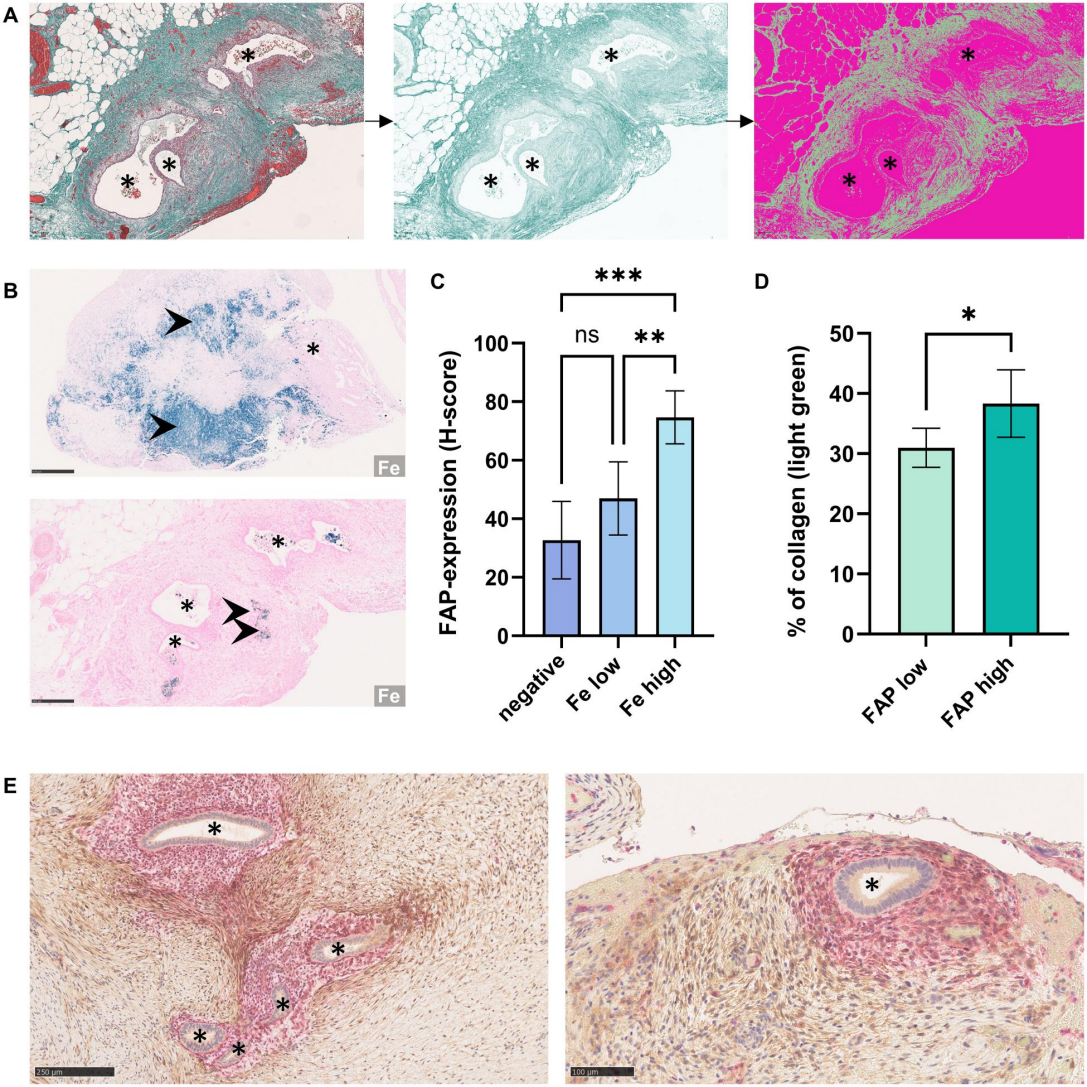
**Analysis of stroma composition in the microenvironment of ectopic endometrium.** (**A**) Analysis of collagen abundance on Masson trichrome staining of peritoneal endometriosis of the pararectal space. Left: original image; middle: light green channel after colour deconvolution; and right: areas above the threshold are classified as positive (sage green) after stain separation and pixel classification. Ectopic endometrioid glands are depicted by asterisks. (**B**) Prussian blue staining for iron (Fe). Strong staining (arrowheads) in deep infiltrating endometriosis of the rectum (top, scale bar: 500 µm) and weak staining (arrowheads) in peritoneal endometriosis of the pararectal space (bottom, Scale bar: 250 µm). Ectopic endometrium is depicted by asterisks. (**C**) Prussian blue staining intensity correlates with fibroblast activation protein-α (FAP) staining. The FAP staining (measured by histoscore [H-score]) of lesions with strong Prussian Blue staining (Fe high) differs significantly from lesions with weak (Fe low) or no staining (negative). (**D**) Samples with increased FAP expression in the stroma within 500 µm of ectopic endometrioid glands (FAP high, H-score ≥100) have a higher percentage of tissue collagen (light green on Masson trichrome staining) than samples with lower FAP expression (FAP low, H-score <100). Data are shown as mean with 95% CI. ns, not significant, *P < 0.05, **P < 0.005, ***P < 0.0005, ordinary one-way ANOVA followed by Dunnett’s multiple comparisons test (C) and two-tailed unpaired *t*-test (D). Comparisons indicated by horizontal brackets. (**E**) Immunohistochemical analysis of FAP and CD10 in deep infiltrating endometriosis (left, scale bar 250 µm) and a superficial peritoneal lesion (right, scale bar: 100 µm). While CD10-positive stromal cells (red) directly surround ectopic endometrioid glands (asterisks), FAP (brown) is also expressed by CD10-negative fibroblasts in greater distance to the glands.

To assess the composition of the stroma in regard to the content of activated fibroblasts, we performed Masson Goldner trichrome staining of consecutive slides followed by digital image analysis to determine the amount of collagen in the tissue. Again, tissue within 500 µm of endometriotic lesions was analysed after annotating the lesions. Pixel classification was then used in the light green channel to determine the percentage of tissue area above the threshold representing collagen (Fig. 2A). We compared the percentage of collagen-positive pixels of samples with higher FAP expression (H-score ≥100) with samples with lower FAP expression (FAP low, H-score <100) and found a significant correlation of collagen-content and FAP expression (mean percentage of collagen-positive tissue 30.97% in FAP low samples vs 38.33% in FAP high samples; *P* < 0.05, Fig. 2D). To determine whether FAP positivity occurs only among the endometriosis-associated CD10-positive cytogenic stroma, we conducted an immunohistochemical double staining for FAP and CD10. This showed that although there is some co-localization of FAP-positive and CD10-positive cell populations in the stroma of endometriotic lesions, FAP expression is not restricted to the cytogenic stroma directly surrounding ectopic endometrioid glands (Fig. 2E).

### Age-dating of lesions

To study factors affecting FAP presence in endometriotic lesions, we explored a connection to the age of the lesion. As implied by Guo *et al.* (2015), the iron content of endometriotic lesions increases over time. Therefore, Prussian Blue staining for iron can be used as an indicator when age-dating endometriotic lesions. We were interested in time-dependent dynamics of fibrosis in endometriosis; therefore, we conducted Prussian Blue staining to study the abundance of tissue iron (Fig. 2B). Iron abundance was classified as either negative, low, or high staining intensity (Fig. 2C). We found a significant difference in FAP expression (H-score) when comparing samples with high iron positivity to negative samples (*P* < 0.0005) or samples with low staining intensity (*P* < 0.005, Fig. 2C), showing that older lesions with higher iron load also show higher FAP expression. There was no significant association of iron deposition and hormonal treatment at the time of surgery (*P* > 0.05).

### Immune microenvironment

To explore the role of the microenvironment of activated fibroblasts surrounding ectopic endometrium, we conducted immunohistochemical analyses of different immune cell populations in this area.

**Figure 3.**
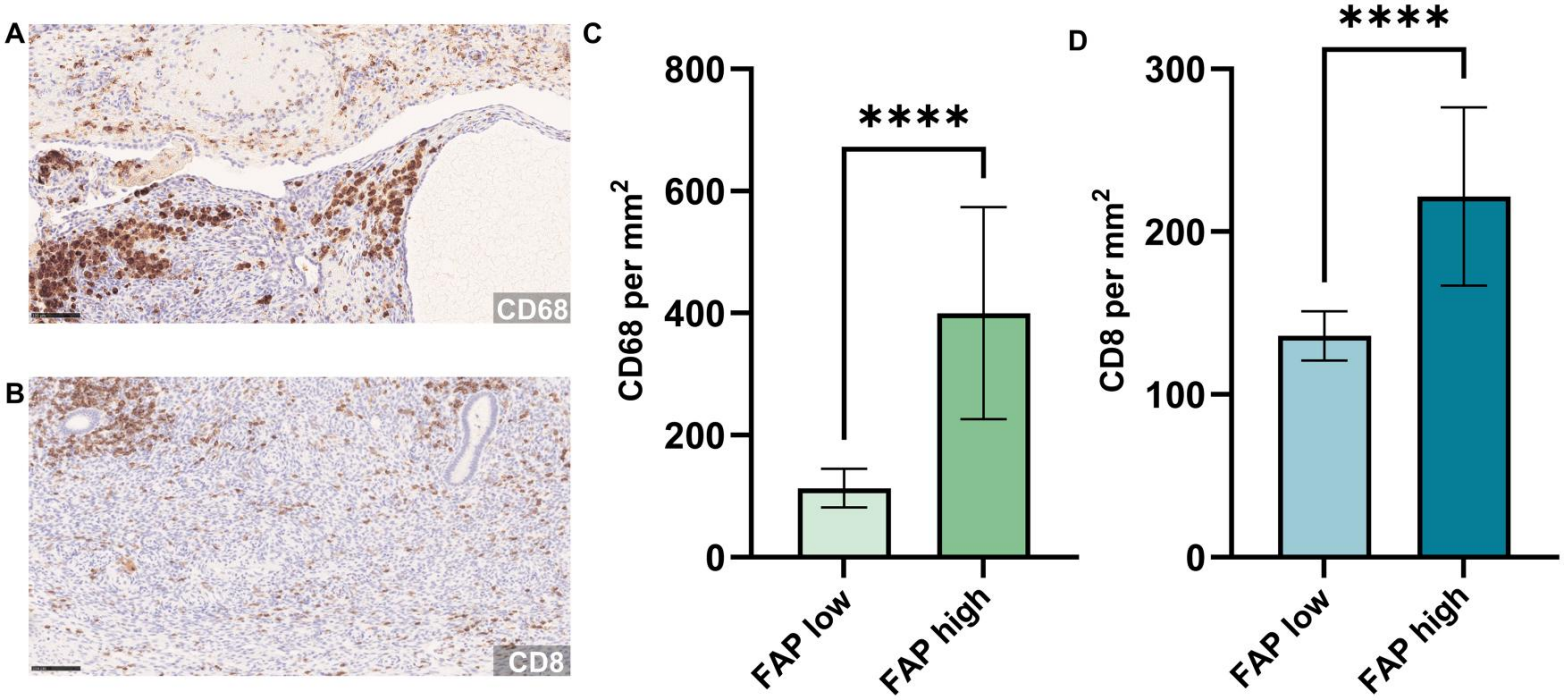
**Analysis of immune cell abundance in lesions with higher or lower fibroblast activation protein-α (FAP) expression.** (**A, B**) Immunohistochemical analysis of CD68 (A) and CD8 (B). Positive cells appear brown. Scale bar: 100 µm. (**C, D**) Samples with increased FAP expression in the stroma within 500 µm of ectopic endometrioid glands (FAP high, histoscore [H-score] ≥100) have a higher percentage of CD68-positive cells (C) and CD8-positive cells (D) than samples with lower FAP expression (FAP low, H-score <100). Data are shown as mean with 95% CI. ****P < 0.0001, two-tailed unpaired *t*-test (C, D). Comparisons indicated by horizontal brackets.

As iron overload contributes to pathological activation of macrophages (Donnez *et al.*, 2016), a cell population highly involved in fibrosis (Chen *et al.*, 2023), we looked into a possible correlation of macrophage abundance and activated fibroblasts. Consecutive tissue slides were immunohistochemically stained with an antibody directed against CD68 to detect macrophages (Fig. 3A). When conducting digital image analysis of CD68-stained slides, we included the interior of the annotation to capture iron-loaded macrophages (i.e. siderophages) within the lumen of ectopic endometrioid glands. We compared samples with higher FAP expression (H-score ≥100) to samples with lower FAP expression (FAP low, H-score <100) and found a higher number of CD68-positive cells in close proximity (within 500 µm) to endometriotic lesions with high abundance of FAP-positive fibroblasts (Fig. 3C).

Both activated fibroblasts and macrophages (Qi *et al.*, 2022) can contribute to T cell exclusion from a tumour microenvironment, and there is evidence that CAFs decrease tumour immunosurveillance by CD8-positive T cells (Herrera *et al.*, 2020). However, T cells in ectopic endometrium are deregulated (Vallve-Juanico *et al.*, 2019). Consequently, we were interested in the abundance of cytotoxic T cells in regard to FAP expression and conducted immunohistochemical analyses of CD8 on consecutive slides to detect cytotoxic T cells (Fig. 3B). When comparing FAP high (H-score ≥100) and FAP low (H-score <100) samples, there was a significant difference (*P* < 0.0001) in CD8 abundance within 500 µm of endometriotic lesions (Fig. 3D). Interestingly, samples with higher FAP expression also had higher numbers of CD8-positive cells per mm^2^.

## Discussion

Endometriosis imposes a tremendous impairment of quality of life. The mechanisms leading to the symptoms such as pain and infertility are not yet fully understood. The interactions of different cell types in the microenvironment are complex. In this study, an objective digital image analysis workflow to analyse the microenvironment of endometriosis was established.

Our results show the presence of activated FAP-expressing fibroblasts in the microenvironment of endometriosis. Although the content of lesions of different locations and types differs, activated fibroblasts were consistently detectable in most samples of superficial peritoneal lesions, ovarian endometriomas, extra-abdominal lesions, and deep infiltrating endometriosis. This indicates that these cells might indeed provide a possible target structure in endometriosis diagnostics. Imaging methods aimed at activated fibroblasts such as FAPi PET/CT might be of interest when exploring future diagnostic options to establish a new diagnosis of endometriosis or when planning surgery for lesion removal to guide surgeons and thereby improve the outcome.

Activated fibroblasts interact with their environment and influence the extracellular matrix composition (Lee *et al.*, 2011; Qi *et al.*, 2022). Our results show that high FAP expression correlates with a higher collagen content of the tissue surrounding endometriotic lesions.

Moreover, we show that FAP expression correlates with tissue iron deposits which increase with the age of the lesion as shown by Guo *et al.* (2015) for endometriotic cysts. These results point to a correlation of the duration of hormonal influence on endometriotic lesions and fibrogenic changes to their environment including fibroblast activation.

The abundance and activation of macrophages are highly intertwined with the composition of the microenvironment of endometriosis (Artemova *et al.*, 2021). In endometriotic lesions, there is evidence that elevated concentrations of heme (which is released by lysed erythrocytes) in the peritoneal fluid of endometriosis patients impair the phagocytotic ability of macrophages (Liu *et al.*, 2019). The continuous delivery of haemoglobin to macrophages leads to iron overload (Defrere *et al.*, 2006) and abnormal macrophage activation, inducing iron-mediated damage and oxidative stress which increases chronic inflammation and drives proliferation and fibrosis of lesions (Donnez *et al.*, 2016; Chen *et al.*, 2023). Moreover, macrophages contribute to extracellular matrix production and fibrosis via the TGFb1/Smad3 signalling pathway and production of fibrogenic mediators (Vigano *et al.*, 2020; Chen *et al.*, 2023). Our results indicate a correlation of the abundance of activated fibroblasts and macrophages in the immune microenvironment of endometriotic lesions. This could be due to fibrogenic capabilities of macrophages inducing fibroblasts activation and extracellular matrix production in lesions with high iron exposure and longstanding hormonal influence.

There is evidence that FAP-positive fibroblasts correlate and interact with SPP1+ macrophages in solid tumours and thereby create an immune-exclusive desmoplastic structure which limits T cell infiltration (Qi *et al.*, 2022). Activated fibroblasts have been associated with impaired immunosurveillance and T cell exclusion in solid tumours (Fearon, 2014; Herrera *et al.*, 2020). We therefore expected either a T cell-exclusive effect of activated fibroblasts or a microenvironment with features of strong chronic inflammation in FAP-high samples. Our results show that activated fibroblasts in endometriosis do not prevent T cell infiltration, as we did not see an immune-exclusive effect of FAP expression, but rather occur in an inflammatory setting positively correlated with the abundance of CD8-positive cytotoxic T cells per mm^2^ in endometriosis. In general, the number of CD8-postive T cells in ectopic endometrium exceeds those of eutopic endometrium (Kisovar *et al.*, 2023) and does not vary with the hormonal milieu as it would in eutopic endometrium; therefore, one can assume an environment of deregulated CD8-positive T cells in endometriotic lesions (Vallve-Juanico *et al.*, 2019). Our results reflect this deregulation and, moreover, indicate a pronounced chronic inflammatory microenvironment (that contributes to fibrosis and adhesions forming) (Chen *et al.*, 2023) in lesions with a high abundance of FAP, and is in line with studies finding deregulated T cells in ectopic endometrium (Vallve-Juanico *et al.*, 2019).

These results lead to the assumption that ongoing hormone-dependent cyclic changes lead to ongoing chronic inflammation and iron deposits. This fuels tissue remodelling and thus fibrosis which is accompanied by increased macrophage and CD8-positive T-cell infiltration. Activated fibroblasts are present in endometriosis and targetable (Kratochwil *et al.*, 2019); therefore, FAPi PET/CT might be a diagnostic option that should be considered for further exploration in the diagnostic workup of endometriosis. To confirm these experimental results, studies comparing staining results with FAPi PET/CT have to be conducted. There are currently promising first approaches to translate these findings into clinical practice by providing endometriosis patients with an FAPi PET/CT prior to surgery for lesion removal (unpublished data), but this has to be validated in a structured way on a larger patient cohort *in vivo*.

## Supplementary Material

hoaf003_Supplementary_Data

## Data Availability

The data underlying this article will be shared upon reasonable request to the corresponding author.
