# Supplementary material for: FAP+ activated fibroblasts are detectable in the microenvironment of endometriosis and correlate with stroma composition and infiltrating CD8+ and CD68+ cells
Source: Hum Reprod Open. 2025 Jan 24;2025(1):hoaf003. doi: 10.1093/hropen/hoaf003 (PMC11829078; doi:10.1093/hropen/hoaf003)
Supplement: hoaf003_Supplementary_Data [file hoaf003_supplementary_data.zip › 2498c_Supplementary table S1.docx]

| **Supplementary table S1**. **Antibodies used for immunohistochemical analyses** | | | | | |  |  |
| --- | --- | --- | --- | --- | --- | --- | --- |
| **Protein** | **Source** | **Catalog No.** | **Species** | **Clonality** | **Dilution** | **pH** | **Buffer** |
| FAP | Abcam Ltd, | ab314456 | rabbit | Multiclonal | 1:300' | 9 | EDTA |
|  | Cambridge, UK | |  |  |  |  |  |
| CD8 | Leica Biosystems Newcastle Ltd, | PA0183 | mouse | Monoclonal | RTU* | 9 | EDTA |
|  | Newcastle Upon Tyne, UK |  |  |  |  |  |  |
| CD68 | Leica Biosystems Newcastle Ltd, | PA0273 | mouse | Monoclonal | RTU* | 9 | EDTA |
|  | Newcastle Upon Tyne, UK |  |  |  |  |  |  |
| CD10 | Leica Biosystems Newcastle Ltd, | PA0131 | mouse | Monoclonal | RTU* | 9 | EDTA |
|  | Newcastle Upon Tyne, UK | | |  |  |  |  |
| *ready to use | |  |  |  |  |  |  |
